# The molecular subtypes of triple negative breast cancer were defined and a ligand-receptor pair score model was constructed by comprehensive analysis of ligand-receptor pairs

**DOI:** 10.3389/fimmu.2022.982486

**Published:** 2022-08-31

**Authors:** Weijun Pan, Kai Song, Yunli Zhang, Ciqiu Yang, Yi Zhang, Fei Ji, Junsheng Zhang, Jian Shi, Kun Wang

**Affiliations:** ^1^ The Second School of Clinical Medicine, Southern Medical University, Guangzhou, China; ^2^ Department of Breast Cancer, Cancer Center, Guangdong Provincial People’s Hospital, Guangdong Academy of Medical Sciences, Guangzhou, China; ^3^ Department of Pathology, Nanfang Hospital, Southern Medical University, Guangzhou, China; ^4^ Department of Pathology, School of Basic Medical Science, Southern Medical University, Guangzhou, China

**Keywords:** triple negative breast cancer, ligand-receptor pairs, tumor microenvironment, drug susceptibility, immunotherapy

## Abstract

**Background:**

Intercellular communication mediated by ligand-receptor interactions in tumor microenvironment (TME) has a profound impact on tumor progression. This study aimed to explore the molecular subtypes mediated by ligand-receptor (LR) pairs in triple negative breast cancer (TNBC), identify the most important LR pairs to construct a prognostic risk model, and study their effect on TNBC immunotherapy.

**Methods:**

LR pairs subclasses of TNBC were categorized by consensus clustering based on LR Pairs in METABRIC dataset. Least absolute shrinkage and selection operator (LASSO) Cox regression and stepwise Akaike information criterion (stepAIC) were conducted to build a LR pairs score model. The relationship between LR pairs score and immune cell infiltration, stromal score and immune score associated with TME was analyzed, and the prediction of drug therapy and immunotherapy efficacy by LR pairs score was evaluated.

**Results:**

According to the expression pattern of 145 TNBC prognostic LR pairs, the samples were divided into three subclasses with different survival outcomes, copy number variation (CNV), TME immune cell infiltration, stromal score and immune score. The LR pairs score model constructed in the METABRIC dataset was composed of four LR pairs, and its predictive significance for TNBC prognosis was verified in GSE58812 and GSE21653 cohorts. In addition, LR pairs score was negatively correlated with several immune pathways regulating immunity and immune score, and related to the sensitivity of anti-neoplastic drugs and the effect of anti-PD-L1 therapy.

**Conclusion:**

Our study confirmed the impact of LR pairs on the molecular heterogeneity of TNBC, characterized three LR pairs subtypes with different survival outcomes and TME patterns, and proposed a LR pairs score system with predictive significance for TNBC prognosis and anti-PD-L1 therapeutic effect, which provides a potential evaluation scheme for TNBC management.

## Introduction

Breast cancer has become the most frequently diagnosed female cancer, accounting for 11.7% of all cancer cases (1). According to the expression of molecular markers of estrogen or progesterone receptors and human epidermal growth factor receptor 2 (HER2), breast cancers are divided into three major subtypes, including hormone receptor positive/HER2 negative subtype (70%), HER2 positive subtype (15%-20%) and triple-negative subtype (tumors lacking all 3 standard molecular markers,15%) (2). Among all three breast cancer subtypes, triple negative breast cancer (TNBC) is the most invasive subtype with the worst prognosis (3). In recent years, a thesis has been put forward that dependent on various clinical, pathological, and genetic factors, triple-negative breast cancer is a separate, heterogenic subtype of breast cancer, (4). Multi-omics profiling studies have provided novel insights into the biological heterogeneity of TNBC, evolutionizing the classification of these tumors into distinct molecular subtypes based on recurrent genetic aberrations, transcriptional patterns, and tumor microenvironment features (5). Here, molecular typing together with the prediction of the prognosis of the gene profile may help to promote the study of personalized treatment.

Tumor is a heterogeneous mixture of cancer cells and non-cancer cells. Communication between these cells within the tumor is the key to tumor progression (6). Communication between these cells is achieved by ligands produced by a cell (proteins, peptides, fatty acids, steroids, gases and other low molecular weight compounds) that are either secreted by cells or present on the cell surface and therefore acts as receptors either on or inside the target cells (7). It is reported that most cells express from tens to hundreds of ligands and receptors, forming a highly connected signal network through multiple ligand-receptor pairs (8). The biological importance and availability of receptors and their corresponding ligands have designated them as particularly useful clinical targets for cancer (9). Therefore, there are broad prospects for the research of ligand-receptor pairs in the field of molecular oncology.

In this study, we analyzed 2293 LR pairs in TNBC. The molecular subtypes of the samples were subdivided by screening LR pairs significantly related to the TNBC prognosis for exploring the heterogeneity of the subtypes defined in relation to copy number variation, tumor immune components and biological pathways. A LR pair score model was constructed by least absolute shrinkage and selection operator (LASSO) COX regression to study its correlation with TNBC prognosis, tumor microenvironment (TME) and clinical treatment response.

## Materials and methods

### TNBC data resources

cBio Cancer Genomics Portal (cBioPortal) is an open-access resource for exploring, visualizing, and analyzing multidimensional cancer genomics and clinical data (10). The METABRIC dataset was downloaded from cBioPortal (http://cbioportal.org/) and screened for availability. Genomic variation data of 318 TNBC samples and the motif table spectrum of 298 samples were obtained. Microarray data of 107 and 83 TNBC samples from GSE58812 and GSE21653 datasets of Gene Expression Omnibus (GEO, https://www.ncbi.nlm.nih.gov/geo/) database were collected.

### Acquisition and screening of ligand receptor pairs

Ligand–receptor (LR) pairs containing 2293 interactions were downloaded from literature-curated database connectomeDB2020. If the sum of gene expression in each pair of LR was equal to or greater than the median of the sum of LR gene expression in all patients, a patient was defined as having a high expression. Otherwise, the patient was defined as having a low expression. The “survival” package in R was used to analyze the correlation between each pair of LR and the survival of TNBC patients in each cohort. The statistical significance was analyzed by the Peto and Peto modification of Gehan-Wilcoxon test, and the exponential coefficient of Cox regression model was develop to calculate the risk ratio (HR). The “sump” function in the “metap” package was employed to integrate the P values of the three cohorts using Edgington’s method, and multiple test corrections based on Storey Method were performed by the “qValue” package. LR pairs with Storey’s q-value < 0.2 and HR > 1 (or HR < 1) was considered to be related to the prognosis of TNBC.

### Establishment of LR subtypes using consensus clustering

Clusters were classified using “ConsensusClusterPlus” based on the expression of TNBC prognosis related LR pairs. The K-means algorithm and “1-Pearson correlation” were specified, and each sample was divided into up to k groups by the clustering algorithm. Each of the bootstraps involved 80% of the samples with 500 repeats. The heat map of consensus clustering was generated by R packet “pheatmap”. The number of clusters was decided by Consensus cumulative distribution function (CDF) plot and delta area plot, and the standard was that the consistency within the cluster was high, the coefficient of variation was low and the area under the CDF curve would not increase significantly.

### Analysis of mutations and copy number variation among subtypes

Genomic data types integrated by cBioPortal include somatic mutations, copy number alterations, gene expression and DNA methylation (11). The study directly inquired and downloaded the somatic mutations and copy number alterations data from cBioPortal, and analyzed them according to the procedures used in the study by Gao et al. (12). The “maftools” software package was used to visualize mutation data. The differences of CNV genes with significant gain and loss subtypes were compared employing chi-square test.

### Functional enrichment analysis

Hallmark Gene sets were retrieved and downloaded from the Molecular Signatures Database (MSigDB) (13). The GSEA analysis of LR clusters was carried out using GSEA software program, and the most significantly enriched signaling pathways were selected derived from normalized enrichment scores (NES), the standard was false discovery rate (FDR) of <0.05.

### Analysis of immunity

Immune score and stromal score were calculated in R package “ESTIMATE” (14) by using expression signatures to infer the ratio of matrix to immune cells in tumor samples. A higher score pointed to a higher content in TME. The infiltration degree of 22 immune cells in TNBC was quantified by CIBERSORT algorithm (15).

### Construction of risk model based on LR pairs

Important genes were screened from LR pairs related to prognosis to construct a risk model. First of all, the prognosis-related LR pairs was analyzed by LASSO penalty Cox regression analysis, which eliminated unimportant LR pairs through reducing the weight of the model parameters. The rest of the LR pairs was filtered through the stepAIC strategy in MASS package. Genes with the lowest stepAIC value were used to build LR pairs score model. The coefficient of each gene was obtained by multivariate Cox regression analysis.

### The significance of LR pairs score model in predicting clinical treatment response

The relationship between LR pairs score and gene expression level in immune checkpoints was determined by Wilcoxon test, and a box diagram was generated for visualization. Tumor Immune Dysfunction and Exclusion (TIDE) (16) predicted the immune checkpoint blockade (ICB) treatment response of the samples through simulating the accurate gene signature of two immune escape mechanisms. We downloaded drug sensitivity data for approximately 1000 cancer cell lines from Genomics of Cancer Drug Sensitivity (GDSC) (http://www.cancerrxgene.org) (17), which is the largest public resource for information on drug sensitivity in cancer cells and molecular markers of drug response. We analyzed breast cell line, including a total of 50 cell lines treated with 190 drugs.Regarding the area-under-curve (AUC) values of the anti-tumor drugs in cancer cell lines as the drug response index, we used Spearman correlation analysis to calculate the correlation between drug sensitivity and LR.score, and the adjusted FDRs were calculated using the Benjamin and Hochberg method. The correlations with | Rs | > 0.2 and FDR < 0.05 were considered as statistically significant ones. Additionally, the half-maximal inhibitory concentration (IC50) values of the recommended antineoplastic drugs Paclitaxel, Veliparib, Olaparib and Talazoparib for TNBC treatment in different LR pairs score groups were compared using pRRophetic package in R.

### Statistical analysis

The statistical data of this study were analyzed by R 4.0.2 software. The Kaplan-Meier survival curve and receiver operating characteristic (ROC) curves were visualized by the “survminer” package and “timeROC”, respectively. LR score and clinical parameters were included in Cox proportional hazard regression to determine independent factors for predicting the prognosis of TNBC. And the p value cutoff was set to 0.05.

## Results

### Screening of LR pairs related to prognosis

**Figure 1:**
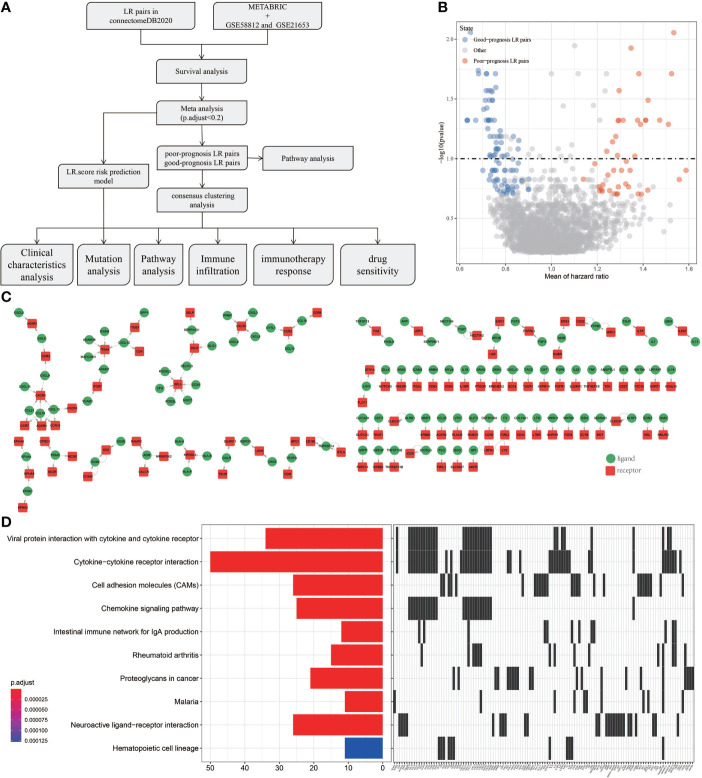
Screening of LR pairs related to prognosis. **(A)** Outline of the process for this study. **(B)** Prognostic volcano maps of 145 LR pairs. **(C)** The interactive network diagram of 145 LR pairs. **(D)** 10 most highly enriched KEGG pathways of 145 LR pairs.

Outline of the process for this study was shown in **Figure 1A**. To screen the LR pairs related to the prognosis of TNBC, survival analysis of LR pairs was performed on METABRIC, GSE58812 and GSE21653. The prognostic significance p-values of the LR pairs resulted from the three cohorts were combined, subjected to meta-analysis, the “sump” function in the “metap” package was employed to integrate the P values of the three queuecohorts through using Edgington’s method, and multiple test corrections based on Storey Method were performed by the “qValue” package. and were subsequently adjusted for multiple testing. A total of 145 LR pairs related to prognosis of TNBC were screened, of which 44 were poor-prognosis LR pairs and 101 were good-prognosis LR pairs (**Figure 1B**). For all the LR pairs related to prognosis of TNBC, we also present the interaction network diagram of them. (**Figure 1C**) and incorporated them into KEGG for pathway further enrichment analysis. Viral protein interaction with cytokine and cytokine receptor, cytokine−cytokine receptor interaction, cell adhesion molecules (CAMs), chemokine signaling pathway, intestinal immune network for IgA production, rheumatoid arthritis, proteoglycans in cancer, malaria, neuroactive ligand−receptor interaction and hematopoietic cell lineage were the 10 most highly enriched pathways of 145 LR pairs (**Figure 1D**).

### Recognition of three TNBC subtypes based on LR pairs

**Figure 2:**
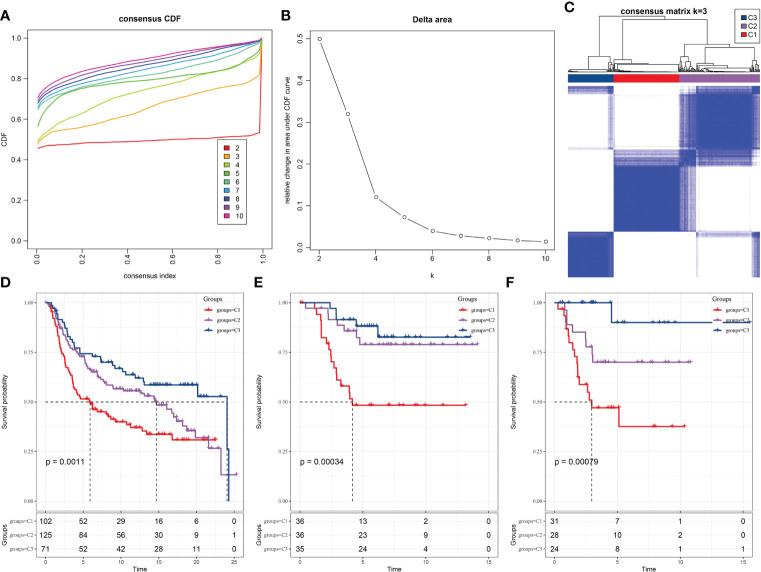
Recognition of three TNBC subtypes based on LR pairs. **(A)** Consensus clustering cumulative distribution function (CDF) for k = 2–9. **(B)** Delta area curve of consensus clustering for samples in METARIC. **(C)** Heatmap of sample clustering at consensus k = 3. **(D)** Kaplan-Meier analysis of OS among three subtypes in METARIC dataset. **(E)** The Kaplan-Meier curve of OS of three molecular subtypes formed in GSE58812 data set. **(F)** Differences of three subtypes in GSE21653 dataset on OS.

We examined whether the TNBC samples can be clustered into subtypes based on the diversity among their expression pattern of the prognosis-related LR pairs. Hence, the significant prognosis-related LR pairs were included as the pattern for clustering, in which the expression abundance of each LR pair was represented by the expression sum of the ligand and receptor genes. In the METARIC cohort, 298 TNBC samples were clustered by ConsensusClusterPlus. And in optimization of the number of clusters, k, the curves of the cumulative distribution function (CDF) suggested that k=3 yielded a stable clustering result (**Figures 2A, B**) and was therefore chosen as the final option (**Figure 2C**). Further analysis of the prognostic characteristics showed significant distinction in prognosis among the three subtypes. The overall survival (OS) of C1 was the most unfavorable, the OS of C3 was the longest of the three subtypes, and the OS of C2 was between the two subtypes (**Figure 2D**). Additionally, we applied the same molecular subtyping method on the TNBC patient cohort of GSE58812 and GSE21653, three molecular subtypes were also formed, and significant and similar difference in prognosis among the three subtypes in survival analysis were observed (**Figures 2E, F**).

### Clinical characteristics and genomic alteration of the LR pairs-based molecular subtypes

**Figure 3:**
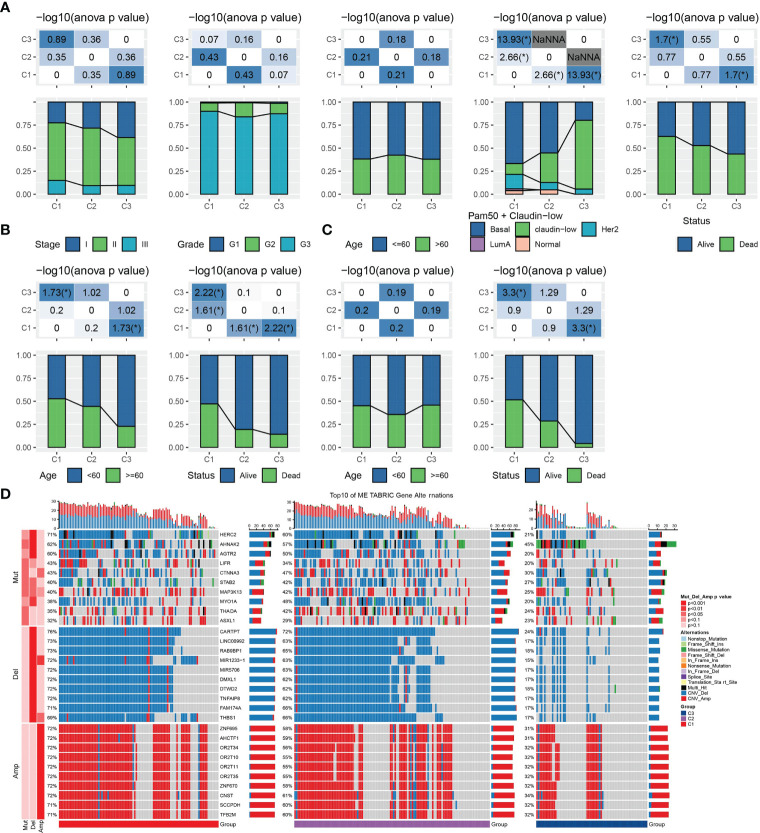
Clinical characteristics and Genomic alteration of the LR pairs-based molecular subtypes. **(A)** The distribution proportion of stage, grade, age, PAM50+claudin-low molecular subtypes and survival status in each subtype of METARIC database. **(B)** The distribution proportion of age and survival status of each subtype in the GSE588123 cohort. **(C)** The distribution of age and survival status among the three subtypes in GSE21653 data sets. **(D)** Waterfall map of somatic mutation and CNV in three subtypes of METARIC database in we had assigned, chi-square test. A symbol "*" indicates ANOVA p < 0.05.

Different clinical features and genomic mutations may also be influencing factors for different prognostic outcomes. We analyzed the clinical characteristics of each subtype in the three TNBC data sets. But no significant correlation was found between the molecular subtypes and clinical variables in METARIC database, such as tumor stage, age and gender. And we noticed significant variation in the distribution of the widely accepted 5 intrinsic molecular subtypes of breast cancer (Luminal A, Luminal B, HER2-enriched, Basal-like and Claudin-low) among the three LR pairs-based subtypes, in which the claudin-low subtype samples accounted for a large proportion of the C3 subtype, and the basal subtype samples accounted for a large proportion of the C1 subtype. There was also a significant difference in mortality between C1 and C3. More than 60% of C1 samples were dead, and more than 55% of C3 samples survived (**Figure 3A**). In the GSE58812 cohort, the age distribution of C1 and C3 had the opposite trend. More than half of the samples in C1 were aged 60 years or older, and more than 75% of the samples were aged under 60. There were also statistically significant differences in survival status among the three subtypes (**Figure 3B**), but there was no significant difference in age distribution among the three subtypes in GSE21653 data set. However, the proportion of survival patients in C1 and C3 was very different, and a high proportion of survival samples were in C3 (**Figure 3C**). The top 10 genes with the greatest variation among the subtypes were displayed as a waterfall plot, and top 10 CNV deletion genes and CNV amplification gens in this heatmap revealed the relatively high mutation rate and mutation diversity in C1 and C2 (**Figure 3D**).

### Functional analysis among the LR pairs-based molecular subtypes

**Figure 4:**
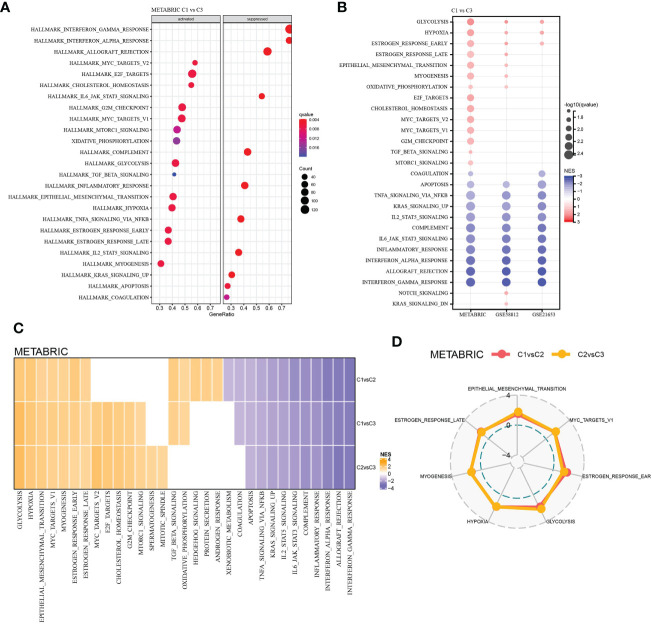
Functional analysis among the LR pairs-based molecular subtypes. **(A)** Bubble chart showing results of the GSEA comparing the C1 with the C3 subtype in METABRIC cohort. **(B)** Bubble chart showing results of the GSEA comparing the C1 with the C3 subtype in the three cohorts. **(C)** Heatmap of the normalized enrichment scores (NES) of the GSEA comparing C1 versus C2, C1 versus C3, and C2 versus C3, and the vertical axis represents the different comparison, while the honrizontal axis represents names of the pathways. **(D)** Radar plot showing pathways coherently activated in C1 versus C2 and C2 versus C3 in the METABRIC database.

To explore the molecular-biological differences between LR pairs-based three molecular subtypes, GSEA was carried out in three TNBC datasets studied. For the GSEA of METARIC database, it was found that compared with C3, 14 pathways in C1 had significantly increased activity, which were largely cell cycle-related signaling pathways such as MYC targets, E2F targets, G2M checkpoint and cancer-related pathways such as glycolysis, hypoxia, etc. And the activity of 11 pathway decreased significantly, which were mainly immune-related pathways such as complement, inflammatory response, interferon alpha response, allograft rejection, interferon gamma response, etc. (**Figure 4A**). In C1 versus C3 of three TNBC datasets, glycolysis, hypoxia and estrogen response early were significantly up-regulated, while 10 pathways, including apoptosis, TNFA signaling *via* NF κ B and complement, were significantly down-regulated (**Figure 4B**). The activity of various pathways was also compared between C1 and C2 and between C2 and C3 subtypes in the METABRIC cohort, and 6 pathways, including glycolysis, hypoxia, epithelial-mesenchymal transition, MYC targets, myogenesis, estrogen response early and late, were activated in each LR pairs-based molecular subtype (**Figures 4C, D**).

### Immune cell infiltration and immune score among the LR pairs-based molecular subtypes

**Figure 5:**
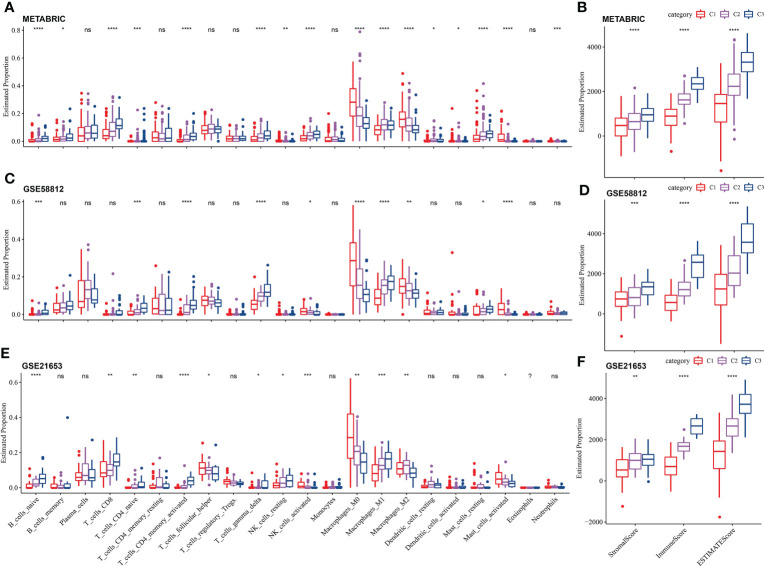
Immune cell infiltration and immune score among the LR pairs-based molecular subtypes. **(A)** The estimated proportion of 22 immune cells among the LR pairs-based molecular subtypes in METABRIC **(A)**, GSE58812 **(C)**, GSE21653 **(E)** cohort. The comparison of stromal score and immune score and ESTIMATE score among three LR pairs-based molecular subtypes in METABRIC **(B)**, GSE58812 **(D)** and GSE21653 **(F)** cohorts calculated by ESTIMATE. P value is calculated by Kruskal-Wallis test, the asterisks represented the statistical p value, ns(no significance), p > 0.05, *p < 0.05, **p < 0.01, ***p < 0.001, ****p < 0.0001.

After running CIBERSORT, we acquired 22 immune cell estimated proportion of three LR pairs-based molecular subtypes in three TNBC cohorts. Kruskal-Wallis test showed that most of immune cells (16 cells in total) with estimated proportion difference among the three LR pairs-based molecular subtypes were in the METABRIC cohort, including naive B cells, memory B cells, CD8 T cells, naive CD4 T cells, activated CD4 memory T cells, delta gamma T cells, resting and activated NK cells, M0 macrophages, M1 macrophages, M2 macrophages, resting dendritic cells, activated dendritic cells, resting and activated mast cells, neutrophils (**Figure 5A**). Naive B cells, naive CD4 T cells, activated CD4 memory T cells, delta gamma T cells, activated NK cells, M0 macrophages, M1 macrophages, M2 macrophages and activated mast cells had significant differences in estimated proportion among LR pairs-based molecular subtypes of all the three TNBC cohorts (**Figures 5C, E**). The stromal score, immune score and ESTIMATE score calculated by ESTIMATE algorithm were compared among subtypes by Kruskal-Wallis test. The immune score showed significant differences among the three molecular subtypes in each cohort, with p values all <0.01. The immune score/ESTIMATE score among the three molecular subtypes in each cohort also showed highly significant differences, with p values all <0.0001. And in whichever of the three scores, C3 was always > C2 > C1 (**Figures 5B, D, F**).

### Construction and evaluation of LR pairs score model

**Figure 6:**
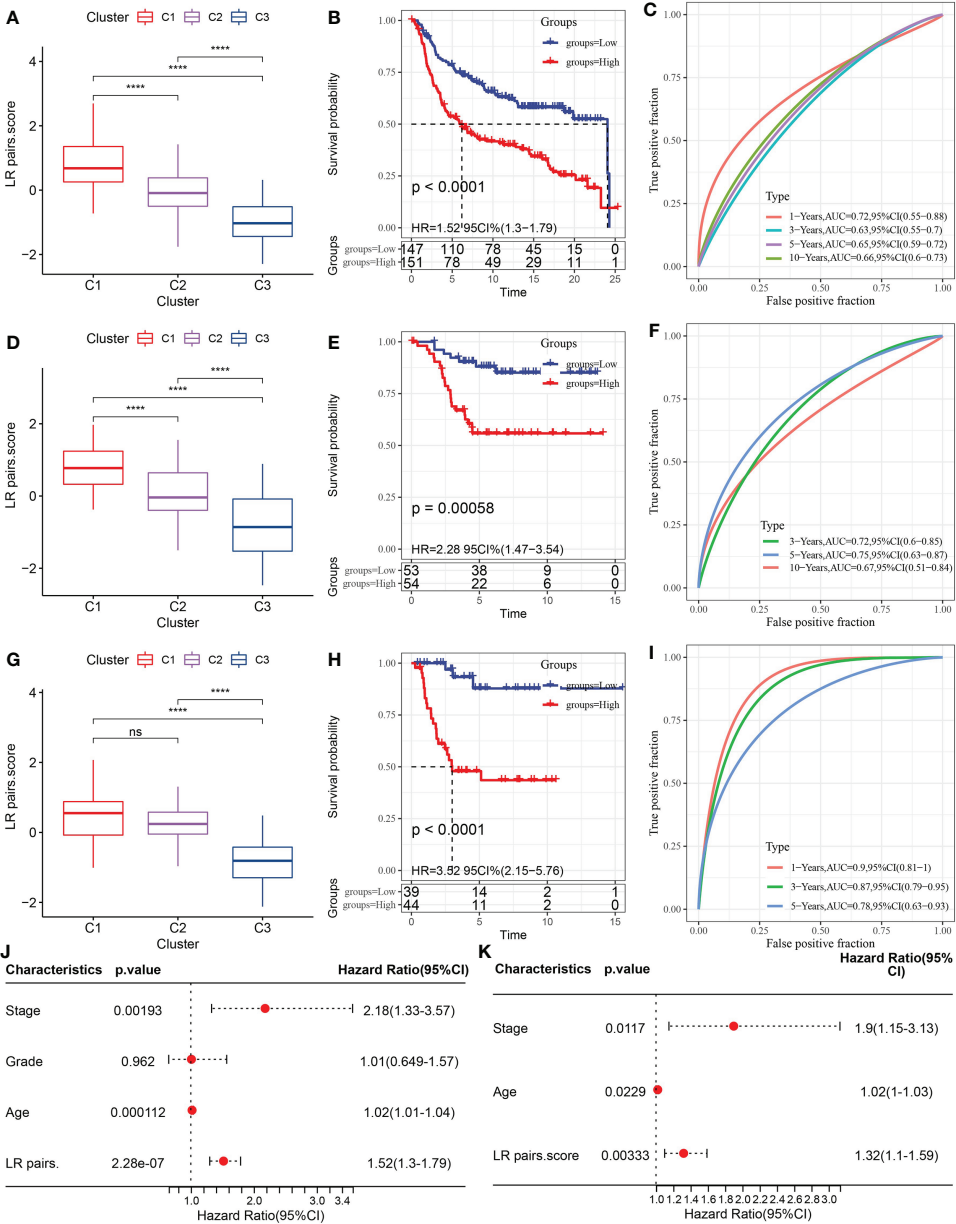
Construction and evaluation of LR pairs score model. **(A)** The box chart of LR pairs scores in three LR pair-based subtypes in the METABRIC cohort, Kruskal-Wallis test. **(B)** Kaplan–Meier estimates comparing OS of samples with distinct LR pairs score in the METABRIC cohort, Log rank test. **(C)** The time-dependent ROC curves showing the prognosis-predicting capacity of LR pairs score in the METABRIC cohort. **(D)**: The box chart showing LR pairs scores in different LR pair-based subtypes in the GSE58812 cohort, Kruskal-Wallis test. **(E)** Kaplan–Meier analysis of the LR pairs score model in the GSE5881cohort, Log rank test. **(F)** The time-dependent ROC curves showing the prognosis-predicting value of LR pairs score model in the GSE58812 cohort. **(G)**. The box chart of LR scores in different LR pair-based subtypes in the GSE21653 cohort, Kruskal-Wallis test. **(H)** Kaplan–Meier estimates comparing OS of samples with distinct LR pairs score in the GSE21653 cohort, Log rank test. **(I)** The ROC curves showing the prognosis-predicting capacity of LR score in the GSE21653 cohort. **(J, K)** The forest plots showing the coefficients and their confidence interval of the univariate and multivariate COX regression which included the factors of LR pairs score, patient age, stage, grade, and patient outcomes in the METABRIC. The asterisks represented the statistical p value, ns(no significance) ****p < 0.0001.

To select the LR pairs the most suitable for predicting the prognosis of TNBC, LASSO COX regression analysis was performed on 145 LR pairs in the METABRIC dataset, and 6 LR pairs were screened in the process of 10-fold cross-validation, as they presented non-zero coefficients in the fitted LASSO COX regression models (**Figure S1A**). Four LR pairs (CXCL9->CCR3, GPI-> AMFR, IL18->IL18R1, and PLG->F2RL1), which had both the statistical fit of the model and the number of parameters used to fit into account, were finally selected by stepwise multifactor regression analysis. The coefficients corresponding to these predictors in the resulted COX regression model were listed in Figure S1B. Based on the 4 LR pairs, an LR-pairs score model, LR-pairs score, was constructed to quantitatively analyze the LR-pairs patterns of TNBC samples. We found that the LR score of the C1 subtype was significantly higher than those of the subtypes C2 and C3 in METABRIC, GSE58812 and GSE21653 cohorts (**Figures 6A, D, G**). To analyze the clinical correlation of LR pairs, the TNBC samples of each cohort were divided into two groups according to LR pairs score. Patients with low LR scores in the METABRIC cohort showed a significantly favorable survival outcome (**Figure 6B**). The area under curve (AUC) of the time-dependent ROC curves of LR pairs score were 0.72, 0.63, 0.65, and 0.66 at 1, 3, 5, and 10 years, respectively (**Figure 6C**). The reliability of LR pairs score was further verified using 107 samples from GSE58812 and 83 samples from GSE21653. In both verification sets, the samples with high LR pairs score showed higher mortality and shorter survival time (**Figures 6E, H**). The AUC values of the LR pairs score model in the GSE58812 validation set were 0.72, 0.75, 0.67 at 3, 5, 10 years, respectively (**Figure 6F**). The LR pairs score model had the optimal performance on another verification cohort GSE21653, with AUC corresponding to 1, 3, and 5 years of survival of 0.90, 0.87, and 0.78, respectively (**Figure 6I**). Also, Univariate Cox regression model analysis in METABRIC showed that stage and age and LR pairs score were significantly correlated with the prognosis of TNBC (**Figure 6J**). These prognostic factors were included in the multivariate Cox regression model, and it was found that they could be regarded as independent prognostic factors of TNBC (**Figure 6K**).

### Correlation between LR pairs score and immune composition and immune-related pathways

**Figure 7:**
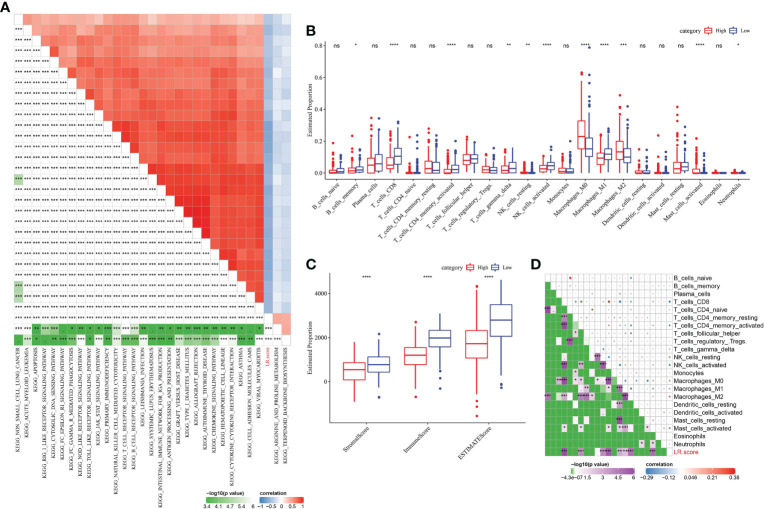
Correlation between LR pairs score and immune composition and immune-related pathways. **(A)** Pearson correlation analyses results between ssGSEA scores of KEGG pathways and LR score in METABRIC with |r|>0.4. **(B)** The box chart showing the relative abundance of the 22 immune cells in high- and low-LR pairs score groups in METABRIC cohort, Wilcoxon test. **(C)** The box chart showing ESTIMATE immune scores of high- and low-LR pairs score groups in METABRIC cohort, Wilcoxon test. **(D)** Pearson correlation analysis of LR pairs score and immune cell components. The asterisks represented the statistical p value, *p < 0.05, **p < 0.01, ***p < 0.001, ****p < 0.0001, ns(no significance).

To find out the most relevant pathway to LR pairs score, R package “GSVA” was used to obtain single sample GSEA (ssGSEA) score of samples in METABRIC with different functions, and 30 pathways significantly related to LR pairs score were obtained by Pearson correlation analysis. Among them, 2 pathways were positively correlated with LR pairs score, while 28 pathways were negatively correlated with LR pairs score. As ssGSEA scores of immune-related pathways, such as chemokine signaling pathway, antigen processing and presentation, natural killer cell mediated cytoxicity, toll like receptor signaling pathway, natural killer cell mediated cytotoxicity and T cell receptor signaling pathway, were significantly negatively correlated with LR pairs score (**Figure 7A**), we further analyzed the relationship between LR pairs score and tumor immune components. Half of the 22 kinds of immune cells were significantly different between high LR pairs score and low LR pairs score samples (**Figure 7B**). We also find high-and low-LR pairs score groups have obvious gap in ESTIMATE and immune scores, and this gap is statistically for all three scores (**Figure 7C**). Furthermore, the Pearson correlation analysis between LR pairs score and immune cells showed that LR pairs score was significantly negatively correlated with CD8 T cells, activated CD4 memory T cells and macrophages, but positively correlated with M0 macrophages and M2 macrophages (**Figure 7D**). These results indicated the association between LR pairs score and tumor immunity.

### Evaluation of the significance of LR pairs score model in the prediction of clinical treatment response

**Figure 8:**
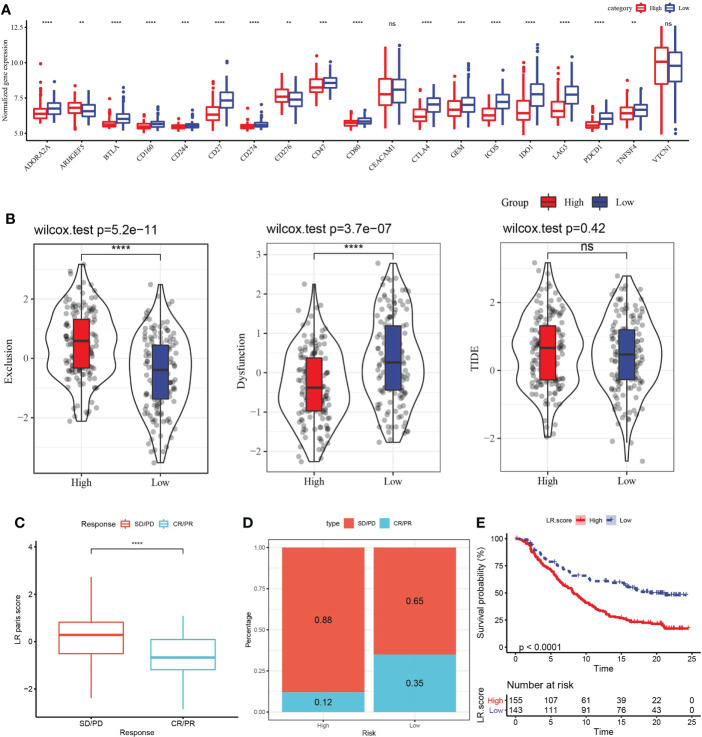
Evaluation of the relationship between LR pairs score model and ICI treatment. **(A)** The association between LR pairs score and gene expression of immune checkpoints, Wilcoxon test. **(B)** The correlation between LR pairs score model and exclusion score, dysfunction score and TIDE score predicted by TIDE method, Wilcoxon test. **(C)** LR pairs score statistical difference between complete response (CR)/partial response (PR) group and stable disease (PD)/progressive disease (PD) group in IMvigor210 cohort. **(D)** The survival curve of different LR pairs score groups in the IMvigor210 cohort. **(E)** Response to anti-PD-L1 treatment in patients with different LR pairs score in the IMvigor210 cohort, Log rank test. The asterisks represented the statistical p value, ns(no significance) **p < 0.01, ***p < 0.001, ****p < 0.0001.

In view of the above association between LR pairs score and tumor immunity, we further analyzed the association between LR pairs score and immune checkpoint genes. In terms of expression, 18 of the 19 immune checkpoints showed differences between the two LR pairs score groups, and the high LR pairs score group had a greater response (**Figure 8A**). The high-LR-pairs-score group also showed significantly up-regulated T cell exclusion score and significantly down-regulated T cell dysfunction score in comparison with low-LR-pairs-score group, while TIDE score showed no significant difference between the two groups (**Figure 8B**). The ability of LR pairs score to predict the response to immune checkpoint inhibitors (ICI) treatment was examined in the immunotherapy cohort IMvigor210 (anti-PDL1). Compared with the samples of complete response (CR) and partial response (PR), the samples of stable disease (SD) and progressive disease (PD) had significantly higher LR pairs score (**Figure 8C**). The samples treated with anti-PD-L1 were divided into low LR pairs score group and high LR pairs score group. In the IMvigor210 cohort, the prognosis of samples with high LR pairs score was still significantly worse than those samples with low LR pairs score (**Figure 8D**). The proportion of patients with low LR pairs scores who responded actively to anti-PD-L1 treatment was significantly more than those with high LR pairs scores (**Figure 8E**).

**Figure 9:**
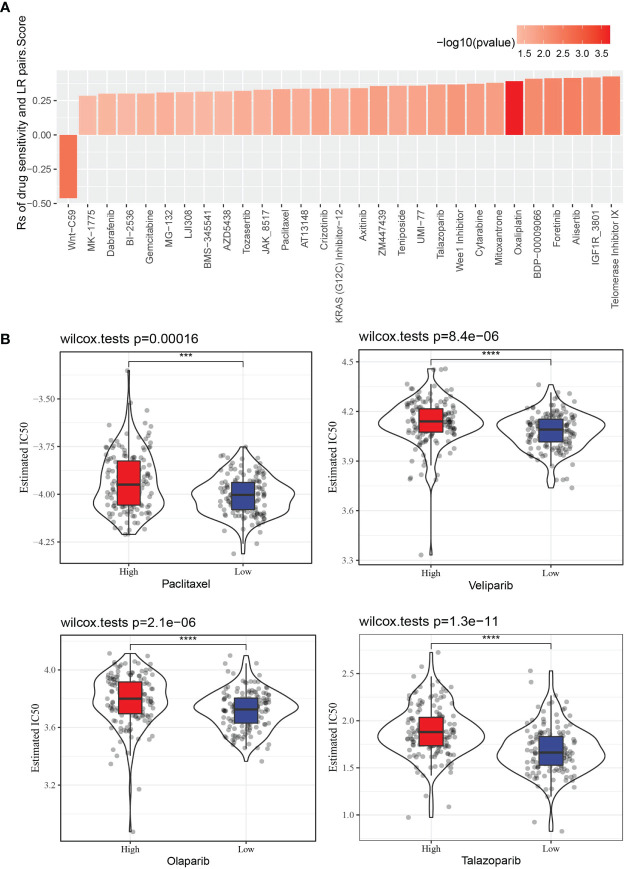
The relationship between LR pairs score and drug sensitivity. **(A)** The correlation between LR pairs score and AUC of drug-sensitive curve, Spearman correlation analysis. **(B)** The violin plot dispalys the differences in the estimated IC50 values of Paclitaxel, Veliparib, Olaparib, Talazoparib between distinct LR pairs score groups, wilcoxon test. The asterisks represented the statistical p value, ***p < 0.001, ****p<0.0001.

The GDSC database stores treatment response data of a wide range of anti-cancer drugs, and gene expression profiles of a large collection of cancer cell lines. Through Spearman correlation analyses of the GDSC data, we found that LR pairs score was significantly correlated to treatment responses of 29 drugs as represented by area-under-curve (AUC) of the drug sensitivity curve. And 28 of the correlation pairs were positive, suggesting that a high LR pairs score in tumor was related to its resistance to these drugs (**Figure 9A**). Besides, the estimated IC50 values of Paclitaxel, Veliparib, Olaparib and Talazoparib in the two LR pairs score groups were compared. It was found that the IC50 values of the four drugs in the low LR pairs score group were significantly lower than those in the high LR pairs score group, indicating that the low LR pairs score group may be more sensitive to the treatment of the four drugs (**Figure 9B**).

## Discussion

In the progression of cancer, cancer cell-stromal cell crosstalk is orchestrated by a plethora of ligand-receptor interactions to generate a TME that favors tumor growth (18). Intercellular communication through LR pairs in the tumor microenvironment underlie the poor prognosis of multiple cancers, such as pancreatic ductal adenocarcinoma (19) and colorectal cancer (20). Increasing discoveries of receptors and ligands and their interactions has encouraged the integration of the available information on ligand-receptor interactions from many databases to facilitate research (21). ConnectomeDB2020 is a database that integrates 2293 pairs of LR interactions. In this study, we analyzed 2293 LR pairs in the database for TNBC.

Firstly, through TNBC survival analysis on 2293 LR pairs, 145 LR pairs significantly related to the prognosis of TNBC were screened. According to the expression of the 145 LR pairs, three LR pairs subclasses of TNBC were obtained employing unsupervised clustering. Among the three LR pairs subtypes, C1 had the worst prognosis, and the proportion of basal-like subtypede, the most aggressive breast cancer subtype (22), was higher in C1 than in the other two groups, and the highest proportion of deaths among the corresponding clinical features. Furthermore, C1 showed the lowest anti-tumor immune response, such as lower tumor infiltrating lymphocytes (naive B cell, CD 8 T cell, naive CD4 T cell) (23) and stromal score and immune score, and these might be the causes of poor prognosis of subtype C1.

In addition to subtyping TNBC based on 145 LR pairs, Lasso regression and Cox analysis were performed on 145 pairs of LR pairs, and 4 pairs of LR pairs were selected to construct an LR pairs score model. Its prognostic significance was confirmed in both TCGA and two GEO datasets. Compared with the samples with low LR pairs score, the samples with high LR pairs score showed significantly shorter survival time. According to previously published reports, Chemokine signaling pathway promotes the antitumor response of the immune system by recruiting immune cells (24). Antigen processing and presentation play a key role in antitumor immunity as the initiation of adaptive immune response (25). The strength of T cell receptor signaling pathway is a key determinant of T cell-mediated antitumor response (26). Natural killer cell mediated cytoxicity is an important effector mechanism of immune system against cancer (27). Activation of the toll like receptor signaling pathway can be used to enhance immune responses against malignant cells (28). In this study, LR pairs score was not only significantly negatively correlated with chemokine signaling pathway, antigen processing and presentation, T cell receptor signaling pathway, natural killer cell mediated cytoxicity, toll like receptor signaling pathway, natural killer cell mediated cytotoxicity (29) and T cell receptor signaling pathway (30) that mediate antitumor immunity, but also with stromal score and immune score and the infiltration of CD8 T cells, activated CD4 memory T cells and macrophages. Additionally, there was no significant difference in TIDE scores between high and low LR pairs scores, and immune escape may not have a significant effect on LR pairs scores. Considering all these results together, we suggested that TNBC samples with high LR pairs score maight not have strong antitumor immunity.

It is reported that different ligands expressed by cancer cells bind to cell surface receptors on immune cells, trigger inhibitory pathways (such as PD-1/PD-L1) and promote immune cells immune tolerance (31). The ability of 4-LR pairs score to predict the response to immune checkpoint inhibitors (ICI) treatment was examined in the anti-PDL1 cohort. We detected that LR pairs score in patients with disease complete response or partial response was significantly lower than that in patients with stable disease or progressive disease. And the clinical benefit from anti-PD-L1 treatment in the low LR pairs score group was significantly greater than that in the high LR pairs score group, which supported the validity of LR pairs score model in predicting anti-PD-L1 treatment.

Researchers have found that some molecular targeted anti-neoplastic drugs can prevent immunotherapy resistance in cancer. Combining these anti-neoplastic drugs with ICI immunotherapy, it can greatly improve the prognosis of patients rather than applying a single drug therapy (32). In this study, 29 pairs of LR pairs score and drug sensitivity were determined in GDSC database by Spearman correlation analysis, of which 28 pairs of drug sensitivity curves showed a significant positive correlation between AUC and LR pairs score. This indicated that they showed drug resistance related to LR pairs score, and only Wnt-C59 showed sensitivity related to LR pairs score.

## Conclusion

In conclusion, according to the expression profile of LR pairs, TNBC was divided into three LR pairs subtypes, which were considerably different in prognosis, CNV, tumor infiltrating immune cells and immune score. In addition, four LR pairs were selected to construct a risk model, which could potentially predict the response of patients to targeted therapy, chemotherapy and immunotherapy.

## Data availability statement

The original contributions presented in the study are included in the article/**Supplementary Material**. Further inquiries can be directed to the corresponding authors.

## Author contributions

WJP, KS, and YLZ contributed the central idea, analyzed most of the data, and wrote the initial draft of the paper. KW and JS oversight and leadership responsibility for the research activity planning and execution, including mentorship external to the core team. The remaining authors contributed to refining the ideas, carrying out additional analyses and finalizing this paper. All authors contributed to the article and approved the submitted version.

## Funding

This study is supported by grants from National Natural Science Foundation of China (82171898, 82171898,82103093), Guangdong Basic and Applied Basic Research Foundation (2022A1515012277, 2020A1515010346, 2021A1515011570), Science and Technology Planning Project of Guangzhou City (202002030236, 202102021055), Beijing Medical Award Foundation(YXJL-2020-0941-0758) and Deng Feng project of high-level hospital construction (DFJHBF202109). Funding sources were not involved in the study design, data collection, analysis and interpretation, writing of the aitical, or decision to submit the article for publication.

## Conflict of interest

The authors declare that the research was conducted in the absence of any commercial or financial relationships that could be construed as a potential conflict of interest.

## Publisher’s note

All claims expressed in this article are solely those of the authors and do not necessarily represent those of their affiliated organizations, or those of the publisher, the editors and the reviewers. Any product that may be evaluated in this article, or claim that may be made by its manufacturer, is not guaranteed or endorsed by the publisher.
