# Supplementary figures and images for: The molecular subtypes of triple negative breast cancer were defined and a ligand-receptor pair score model was constructed by comprehensive analysis of ligand-receptor pairs
Source: Front Immunol. 2022 Aug 31;13:982486. doi: 10.3389/fimmu.2022.982486 (PMC9470927; doi:10.3389/fimmu.2022.982486)

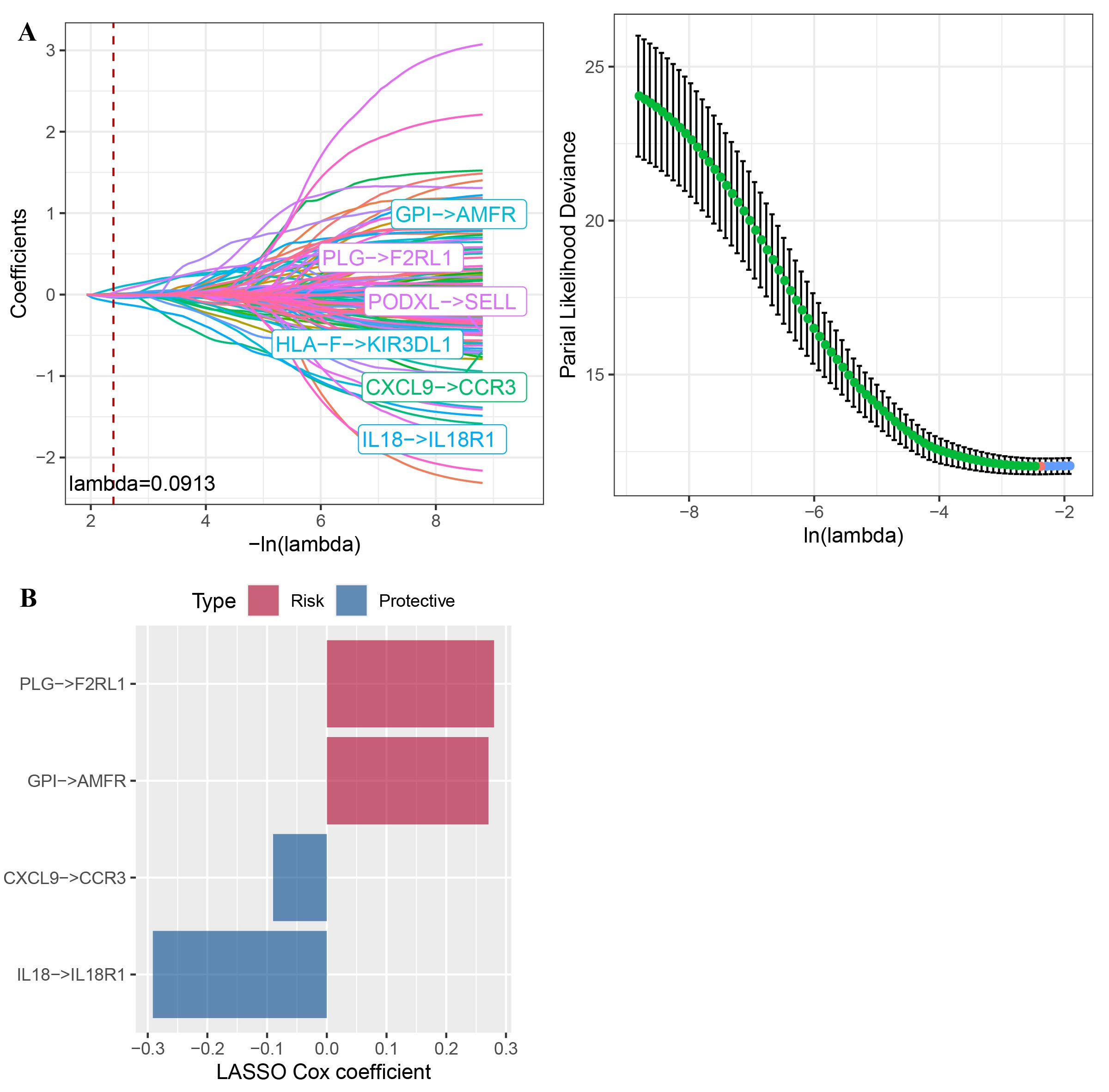

Supplement: Supplementary file 1 [file Image_1.jpeg]
